# Structural Failure of a Modern Knee Tumor Megaendoprosthesis

**DOI:** 10.1155/2017/2429459

**Published:** 2017-12-21

**Authors:** Katrin Koch, Dieter Kohn, Konstantinos Anagnostakos

**Affiliations:** ^1^Klinik für Orthopädie und Orthopädische Chirurgie, Universitätskliniken des Saarlandes, Homburg, Saar, Germany; ^2^Zentrum für Orthopädie und Unfallchirurgie, Städtisches Klinikum Saarbrücken, Saarbrücken, Germany

## Abstract

Modular knee megaendoprotheses are commonly used devices for distal femur or proximal tibia replacement in tumor surgery as well as for treatment of some periprosthetic fractures around a loose or failed total knee arthroplasty. Structural failures of the prosthesis are well-known postoperative complications and have been reported for various prosthesis types. In the majority of the cases, the polyethylene parts fail. We would like to present an unusual case of a broken femoral component of an MRH® endoprosthesis four years after implantation.

## 1. Introduction

Modular knee megaendoprotheses are commonly used devices for distal femur or proximal tibia replacement in tumor surgery [1, 2] but also in the treatment of some periprosthetic fractures around a total knee arthroplasty [1, 3].

Despite innovations in materials and implant design, these implants are associated with a higher complication rate compared with standard endoprostheses [1, 2]. In a retrospective multicenter study, Henderson et al. identified and classified five modes of failure in oncologic patients: soft-tissue failures (type 1), aseptic loosening (type 2), structural failures (type 3), infection (type 4), and tumor progression (type 5) [2]. This classification system was further specified including biological and expandable reconstructions by the International Society of Limb Salvage (ISOLS) [4]. With regard to structural failures, several reports have been published about different tumor prosthesis systems [2, 5–7].

For reconstructive surgery of the knee joint, the Global Modular Replacement System combined with the Modular Rotating Hinge (GMRS®/MRH, Stryker Co.) is an option. Regarding structural failures, literature data show an extremely low rate for this type of prosthesis. In a large study of 247 GMRS prostheses, Pala et al. reported a rate of 29% for types 1, 2, 4, and 5 failures but none for type 3 [8].

We will report an unusual case of a broken femoral component of a GMRS endoprosthesis four years after implantation.

## 2. Case Report

In October 2006, a 21-year-old female patient presented in our department with pain in her right knee. Imaging showed malignant growth in her right proximal tibia with a lateral soft-tissue mass. She was diagnosed with osteosarcoma by open biopsy. CT scans of her chest and abdomen showed no signs of metastases. The patient underwent neoadjuvant chemotherapy in accordance with the EURAMOS 1 protocol.

In January 2007, the patient was surgically treated with a wide resection of the tumor. Surgery included resection of 10 cm of the proximal tibia, 8 cm of the proximal fibula, the M. popliteus, parts of the M. tibialis anterior, M. extensor digitorum longus, and M. extensor hallucis longus. Reconstruction was performed with the GMRS combined with the MRH. The preparation of the distal femur was made in accordance with the surgical technique provided by the manufacturer, required no bone augmentation, and there were no technical difficulties. The patellar tendon was refixed onto the prosthesis with a modified McLaughlin cerclage technique using FiberWire® (Arthrex Inc.) and Mersilene® tape (Ethicon Inc.). For sufficient soft-tissue coverage, a lateral gastrocnemius flap was performed. In order to achieve R0 resection of the tumor, it was not possible to preserve the superficial peroneal nerve, resulting in a peroneal palsy with complete loss of strength for dorsiflexion of the right foot and big toe and loss of sensation on the right dorsum of the foot. Histological examination of the removed tissues confirmed complete resection of the sarcoma (R0, pT1NxMx high-grade sarcoma, regression degree IV Salzer-Kuntschik–Delling—poor responder). Postoperatively, antibiotics were administered for four days. After 4 days of bed rest, the patient was mobilized under non-weight-bearing on crutches for 6 weeks. The right knee joint was immobilized with a knee immobilizing brace for 6 weeks while flexion was allowed up to 70°, first passively and then actively during physiotherapy. 14 days after surgery, the patient continued with the adjuvant chemotherapy following the EURAMOS 1 protocol.

The further postoperative course and the oncological follow-ups were uneventful. There was free range of motion of the right knee joint (extension/flexion 0-0-130°), and the patient reported no impairment of mobility in daily life activities. She remained cancer-free after 5 years.

In March 2011, the patient first reported of pain in her right knee and intermediate swelling on the lateral side of the joint. 3-phase bone scintigraphy showed aseptic loosening of the femoral component. In May 2011, the patient was reoperated. Wear of the tibial polyethylene insert leading to osteolysis of the femoral bone could be identified as a cause of loosening. After removal of the loose femoral component, debridement of the bone replacement was carried out again with the MRH system. Press-fit fixation of a longer offset stem and cement fixation of the condylar component were used. An augmentation of the bone with metal blocks was not necessary. All polyethylene parts of the rotating hinge mechanism were exchanged. The patient was mobilized under non-weight-bearing for 6 weeks after surgery. Regular follow-up examinations in our department showed no abnormalities.

**Figure 1:**
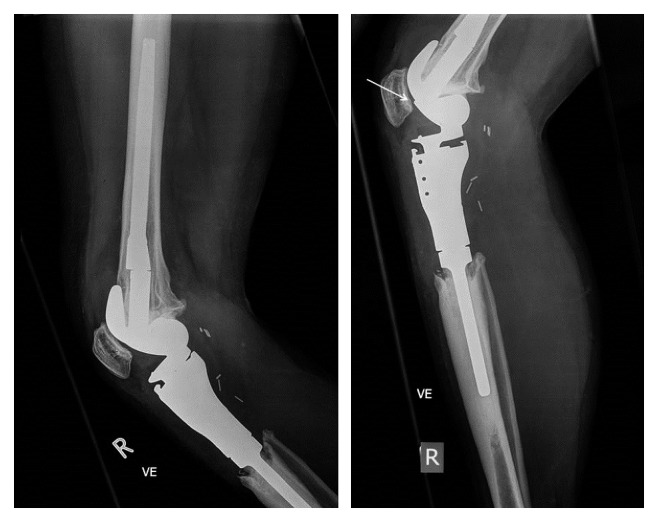
X-rays of the right knee with distal femur and proximal tibia in June 2015. Left (AP view): some bone resorption at the prosthesis-tibia interface. Intact, well-fixed prosthesis. Right: lateral X-rays demonstrated a small step (arrow) of the lateral femur condyle of the prosthesis.

In June 2015, the by-then 30-year-old patient presented with a sudden onset of pain in her right knee. She reported no trauma, and the knee showed no signs of infection. Flexion was reduced to 80°. A bone scan showed no loosening or infection but slightly increased tracer enhancement adjacent to the right lateral femoral condyle. Four weeks later, the patient still reported pain with an additional sensation of temporary locking during flexion and extension. The radiological examination of the knee showed a small step of the lateral femur condyle of the prosthesis in the lateral X-rays (Figure 1).

**Figure 2:**
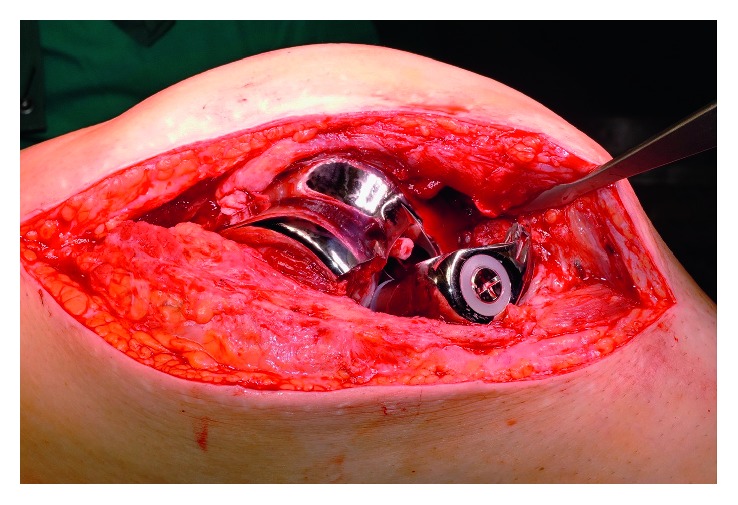
Intraoperative findings showing a fractured lateral condyle of the femoral component.

**Figure 3:**
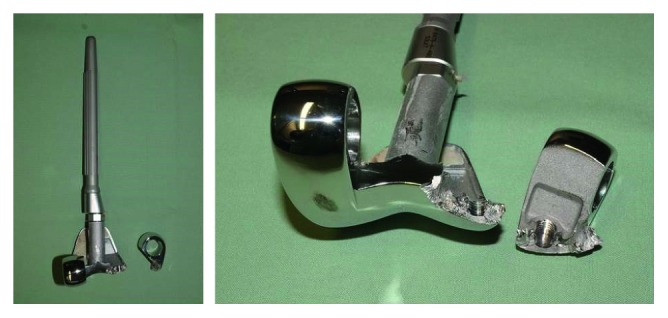
The evaluation of the fractured prosthesis area demonstrated that the breaking line ran through the thread hole where metal augmentation blocks can be added and secured in case of bony defect.

Revision surgery took place in August 2015 and showed breakage of the lateral part of the femoral component (Figure 2). Examination of the damaged femoral component showed that the breaking line ran through the thread hole where metal augmentation blocks can be attached and fixed in case of bony defect (Figure 3). The femoral component was removed and replaced by a new MRH component. The osseous defect in the femoral condyle was augmented by an autologous corticocancellous bone graft and a distal metal block. Immediate full-weight-bearing was allowed, and the patient was discharged with a range of motion of extension/flexion of 0-0-100°. Postoperative follow-ups have so far been uneventful.

## 3. Discussion

The largest number of tumor prostheses including a total of 2174 patients has been evaluated by Henderson et al. [2]. These patients had received partial or total humerus or femur replacement, replacement of the proximal tibia, or a combination of distal femur and proximal tibia replacement. The segmental endoprostheses that were used included the Global Modular Replacement System (GMRS) in 365 patients (17%), the Howmedica Modular Resection System (HMRS®) in 1165 patients (54%), the Kotz Modular Replacement System® in 199 patients (9.2%), and the Modular Replacement System® in 402 patients (19%). Structural failures (type 3) accounted for 17% of all failures and were the highest for distal humeral and distal femoral replacements. Structural failure was the lowest for proximal humeral and total femoral replacements. The rate of structural failure was significantly higher in the uniaxial knee endoprostheses compared with the polyaxial types. Structural failures also occurred more often in the lower extremity compared with the upper extremity. No differentiation was made regarding the rate of structural failures for each prosthesis type.

Structural failures are not an uncommon complication after tumor prosthesis implantation. Agarwal et al. identified 28 breakages in 266 megaprosthetic knee arthroplasties [5]. In the great majority of the cases, the TMH-Nice (Tata Memorial Hospital new indigenous customized prosthesis) was affected. The most common site of breakage was the stem collar junction followed by those in the transverse groove on the stem, those through the body of the implant, and those at the junction of the stem with the tibial base plate. Capanna et al. evaluated 200 Megasystem-C® megaprostheses (Link Co.) in lower limb reconstruction after tumor resection at a minimum follow-up of two years [7]. Structural failures were observed in 7% of the cases including prosthesis breakage, periprosthetic fractures, and articular hinge or polyethylene liner failures. Bus et al. reported a 14% structural failure rate of MUTARS® knee prostheses after a mean of three years, consisting of fractures and unlocking of the locking mechanism, instances of wear, fractures of the femoral component, and stem fractures [6]. Ilyas et al. reported about one femoral stem fracture of a HMRS prosthesis three years after surgery [9]. Heisel et al. observed a 10% failure rate of the polyethylene locking mechanism of MUTARS prostheses at a minimum follow-up of two-years [10]. Biau et al. reported a 10% mechanical complication rate of the GUEPAR® prosthesis including femoral and tibial stem fractures, severe bushing wear, and a hinge fracture [11].

In contrast to other prostheses, literature data show that the GMRS is an established system in the management of periprosthetic fractures after total knee arthroplasty and tumor surgery with a low complication rate. Jassim et al. reported about the use of the GMRS in the management of periprosthetic fractures around total knee arthroplasty in 11 patients and found no mechanical or structural complications at a mean follow-up of 33 months [12]. Sharma et al. evaluated their experience of 77 consecutive distal femoral replacements with the GMRS for limb salvage of tumors [13]. Three patients sustained fractures of their mobile tibial bearing component within four years after implantation, whereby all metal fatigue fractures occurred at the junction of the tibial bearing surface and stem. In a large retrospective study of 247 GMRS prostheses, Pala et al. could observe a failure rate of 29% for type 1, 2, 4, and 5 failures but none for type 3 at a mean follow-up of four years [8].

To the best of our knowledge, this is the first case of a broken femoral component of the GMRS system. Of particular interest is the location of fracture in our case. The breaking line ran through the thread hole where metal augmentation blocks can be attached and fixed in case of bony defect. Since the patient had only a minor bony defect in 2011, no distal augmentation blocks were necessary and these holes were left empty. This leads to the question if the thread holes might be a predetermined breaking point. Another possible explanation might include microfractures of the cement over time or subsequent bone loss that might have weakened the prosthesis-bone interface. After obtaining consent of the patient, the broken femoral component has been sent to the manufacturer who will investigate further the cause of this unique catastrophic failure.
